# The Inhibition of DNA Viruses by the Amphibian Antimicrobial Peptide Temporin G: A Virological Study Addressing HSV-1 and JPCyV

**DOI:** 10.3390/ijms23137194

**Published:** 2022-06-28

**Authors:** Maria Elena Marcocci, Bianka Gabriela Jackowska, Carla Prezioso, Virginia Protto, Marta De Angelis, Francesco Saverio Di Leva, Bruno Casciaro, Alfonso Carotenuto, Maria Luisa Mangoni, Anna Teresa Palamara, Valeria Pietropaolo, Giovanna De Chiara, Lucia Nencioni

**Affiliations:** 1 Department of Public Health and Infectious Diseases, Sapienza University of Rome, Laboratory Affiliated to Istituto Pasteur Italia-Fondazione Cenci Bolognetti, 00185 Rome, Italy; biankagjackowska@gmail.com (B.G.J.); carla.prezioso@uniroma1.it (C.P.); virginia.protto@uniroma1.it (V.P.); marta.deangelis@uniroma1.it (M.D.A.); annateresa.palamara@uniroma1.it (A.T.P.); valeria.pietropaolo@uniroma1.it (V.P.); 2 IRCSS San Raffaele Roma, Microbiology of Chronic Neuro-Degenerative Pathologies, 00163 Rome, Italy; 3 Department of Pharmacy, University of Naples “Federico II”, 80131 Naples, Italy; francesco.dileva@unina.it (F.S.D.L.); alfonso.carotenuto@unina.it (A.C.); 4 Department of Biochemical Sciences, The Istituto Pasteur—Fondazione Cenci Bolognetti, Sapienza University of Rome, 00185 Rome, Italy; bruno.casciaro@uniroma1.it (B.C.); marialuisa.mangoni@uniroma1.it (M.L.M.); 5 Department of Infectious Diseases, Istituto Superiore di Sanità, 00161 Rome, Italy; 6 Institute of Translational Pharmacology, National Research Council (CNR), 00133 Rome, Italy; giovanna.dechiara@ift.cnr.it

**Keywords:** HSV-1, JCPvV, temporins, antimicrobial peptides, antiviral agents

## Abstract

Herpes simplex virus type-1 (HSV-1) and John Cunningham polyomavirus (JCPyV) are widely distributed DNA viruses causing mainly asymptomatic infection, but also mild to very severe diseases, especially when these viruses reach the brain. Some drugs have been developed to inhibit HSV-1 replication in host cells, but their prolonged use may induce resistance phenomena. In contrast, to date, there is no cure for JCPyV. The search for alternative drugs that can reduce viral infections without undermining the host cell is moving toward antimicrobial peptides (AMPs) of natural occurrence. These include amphibian AMPs belonging to the temporin family. Herein, we focus on temporin G (TG), showing that it strongly affects HSV-1 replication by acting either during the earliest stages of its life cycle or directly on the virion. Computational studies have revealed the ability of TG to interact with HSV-1 glycoprotein B. We also found that TG reduced JCPyV infection, probably affecting both the earliest phases of its life cycle and the viral particle, likely through an interaction with the viral capsid protein VP1. Overall, our results are promising for the development of short naturally occurring peptides as antiviral agents used to counteract diseases related to HSV-1 and JCPyV.

## 1. Introduction

Antimicrobial peptides (AMPs) are natural peptides known to be produced in humans, animals, and plants as part of the innate immune response against injury and pathogens. They are short peptides that, in most cases, show high specificity towards pathogens with minimal toxicity to the host. These features make AMPs good candidates for drug development [1,2,3,4].

Amphibian temporins constitute a well-known AMP family with potent antibacterial activity against Gram-negative and Gram-positive bacteria as well as fungi [5,6]. They are mildly cationic and small (10–13 amino acids) peptides secreted from the frog dermal glands to counteract pathogen infections and to control the natural microbial flora [7]. We previously reported the antiviral efficacy of the isoform temporin B (TB) in an in vitro model of herpes simplex virus type 1 (HSV-1) infection where TB was found to disrupt the viral envelope [8]. More recently, we demonstrated that other members of the temporin family affect the life cycle of some enveloped RNA viruses, such as influenza A virus, parainfluenza virus, and severe acute respiratory syndrome coronavirus 2 (SARS-CoV-2), while in some cases, they also alter the integrity of the virions [9,10]. These data suggest the potential of these peptides to display a broad spectrum of antiviral activity that deserves further investigation. 

Herpes simplex virus 1 (HSV-1) is an enveloped double-stranded DNA (dsDNA) virus that generally causes primary infection on the oropharyngeal mucosa, but it can establish latency in neurons mainly of the peripheral ganglia followed by periodic reactivations [11]. Host cell attachment and entry mainly occur via envelope fusion with the plasma membrane, driven by the binding between the viral glycoproteins (gB, gC, gD, and the gH/gL complex, all embedded into the viral envelope) and cellular receptors (e.g., herpesvirus entry mediator, heparin sulfate moieties, nectin1, and nectin2). The incoming tegument protein and viral capsid travel to the nucleus where the viral genome is then released to either start the sequential transcription of viral genes that characterize the productive infection or to establish latency [12]. Reactivation from latency may result either in asymptomatic virus shedding or recurrent herpes diseases [13]. HSV-1 was also reported to contribute to several neurological diseases, including Bell’s palsy, the most typical acute neuropathy of cranial nerves, but not as a determinant etiological element [14]. There is no cure for HSV-1-related diseases, as the virus cannot be eradicated from hosts, but various antiviral medications (acyclovir and derivatives) are available to reduce the severity and frequency of symptoms, likely preventing potential long-term sequelae [15]. While immunocompetent patients can rapidly elicit an efficient immune response to control HSV-1 infection, thus requiring no or short-term antiviral therapy with minimal risk of developing resistance [16], in immunocompromised individuals, HSV-1 recurrences are associated with severe symptoms and, albeit rarely, with serious complications, such as keratitis and encephalitis (HSE) and related sequelae in the central nervous system (CNS) [13]. In these cases, the administration of common anti-HSV-1 drugs can be affected by dose-limiting toxicity, and a prolonged pharmacological treatment can lead to the development of resistant strains [16]. Therefore, the management of viral infections calls for the enlargement of the available drug portfolio.

John Cunningham polyomavirus (JCPyV) is a small non-enveloped double-stranded DNA virus; its circular genome consists of early and late coding regions [17,18] encoding functional [i.e., large T antigen (LTAg) and small t antigen (stAg)] and structural (VP1, VP2, and VP3) proteins, respectively. Between early and late coding regions, there is a non-coding control region (NCCR), which contains the promoter/enhancer elements for the expression of viral genes, the origin of viral DNA replication [19], and the binding sites for host transcriptional factors [20,21]. Notably, its sequence variation determines virus tropism and pathogenicity [22]. The VPs are essential for early events of the virus life cycle, such as the attachment of VP1 pentamers to lactoseries tetrasaccharide c (LSTc) oligosaccharides [23] and the consecutive penetration inside the host cell. Interestingly, VP1 is the most abundant structural protein, with 360 copies, accounting for 80% of the capsid components [24]. Primary JCPyV infection, followed by primary viremia, occurs in childhood, and a life-long asymptomatic and persistent infection is established in the urinary tract, the major site of viral persistence, where JCPyV shedding in urine can occur [19,25,26,27]. When immune control is severely compromised, the virus can reactivate, undergoing secondary viremia, characterized by uncontrolled JCPyV replication and dissemination. Virus spreading to CNS results in a fatal, demyelinating disease known as progressive multifocal leukoencephalopathy (PML) [28]. Specifically, within the brain, JCPyV predominately targets oligodendrocytes and astrocytes [17], causing multiple lesions of demyelination which lead to the development of different clinical symptoms often characterized by motor dysfunction, visual defects, ataxia, and speech impairment [29,30]. Although, to date, advancements in the treatment of immunosuppression and underlying disease have improved survival rates, a cure for the PML disease is still lacking [31]. 

Herein, we evaluate the antiviral activity of temporin G (TG) against HSV-1 and JCPyV in vitro and explore the potential viral targets of TG through the aid of computational studies. We identify HSV-1 gB and JCPyV VP1 as the proteins that can interact with the peptide, thus supposing that TG could work as an antiviral agent by directly targeting the structures of HSV-1 and JCPyV that are essential for the onset of their replicative cycles.

## 2. Results

### 2.1. Temporin G Inhibits the Early Stages of the HSV-1 Replicative Cycle

**Figure 1:**
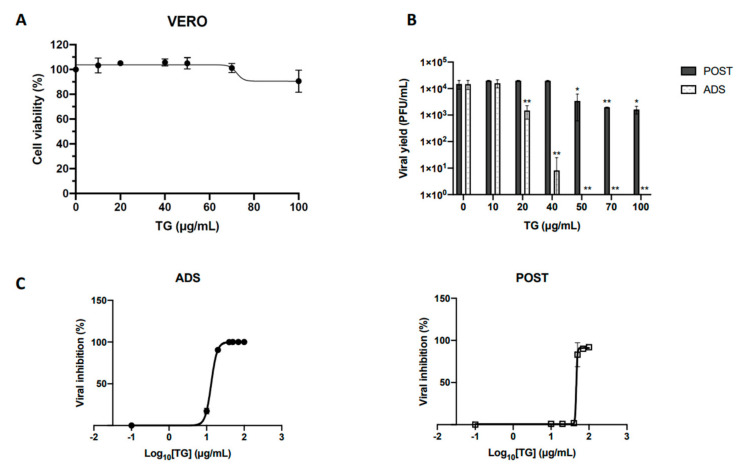
Effects of TG on HSV1 replication in Vero cells. (**A**) Cell monolayers were incubated for 24 h with increasing concentrations of the compound, and cell proliferation was determined by an MTT assay. (**B**) Increasing concentrations of TG (from 0.1 to 100 µg/mL) were added to Vero cells during viral adsorption to host cells [ADS] or after viral adsorption for the following 20 h [POST], and untreated HSV-1-infected cells were used as a comparative control. HSV-1 titers were calculated by a plaque reduction assay and expressed as PFU/mL. (**C**) The antiviral (IC50) effects of TG against HSV-1 were calculated by regression analysis for the ADS (left) and POST (right) conditions. Data are expressed as mean values ± the SD from three independent experiments, each performed in duplicate (*, *p* < 0.05; **, *p* < 0.01; versus the untreated sample; unpaired *t*-test).

To check the antiviral properties of TG against HSV-1, we initially evaluated its cytotoxicity against Vero cells that are highly permissive to HSV-1 infection. To this aim, a Trypan blue exclusion assay was performed, analyzing the viability of cell monolayers treated with increasing concentrations (10–100 μg/mL) of TG for 24 h. A slight decrease in cell viability was observed only in cells treated with the highest TG concentration (100 μg/mL, data not shown). Consistently, the MTT proliferation assay revealed a small reduction of cell proliferation only in samples treated with 100 μg/mL TG (~10% reduction) (Figure 1A). The peptide concentration required to reduce cell viability by 50% (CC50) was found to be 519 μg/mL.

Subsequently, to verify the possible antiviral activity of TG against HSV-1, a time-of-addition assay was performed by focusing on two specific phases of the virus life cycle: the virus attachment/entry into the host cell and the subsequent replication phase into the host cell. For this purpose, Vero cells were infected with 1 multiplicity of infection (MOI) of HSV-1, and increasing concentrations of TG (10, 20, 40, 50, 70, and 100 μg/mL) were administered only during the virus adsorption phase (for 1 h; ADS) or immediately after (and left for 20 h; POST). Untreated HSV-1-infected Vero cells were used under the same conditions as a comparative control. The efficacy of viral infection in TG-treated and -untreated cells was evaluated by measuring the viral titer in cell supernatants harvested 20 h post-infection (PI) by a standard plaque assay (SPA) and estimated as plaque-forming units (PFU/mL). Data in Figure 1B show that the viral titer (i.e., the efficacy of the infection) decreased in cells treated with the peptide during the adsorption phase (ADS). The reduction of viral titer occurred in a TG dose-dependent manner and resulted in about 3 and 4 log reductions when cells were treated with TG at 40 μg/mL and 50 μg/mL, respectively, corresponding to the inhibition of HSV-1 infection greater than 99%. Conversely, TG was less effective in inhibiting virus replication when administered after the adsorption phase (POST), causing only a 1 log reduction at 50 μg/mL of TG (Figure 1B). The 50% inhibitory concentration (IC50) of TG was 13.47 µg/mL and 46.25 µg/mL for ADS and POST treatment, respectively (Figure 1C, left and right panels). The selectivity index (SI), calculated as CC_50_/IC_50_, was 38.5 (for ADS) and 11 (for POST). Based on these results, the next experiments were performed using TG at 50 µg/mL. 

**Figure 2:**
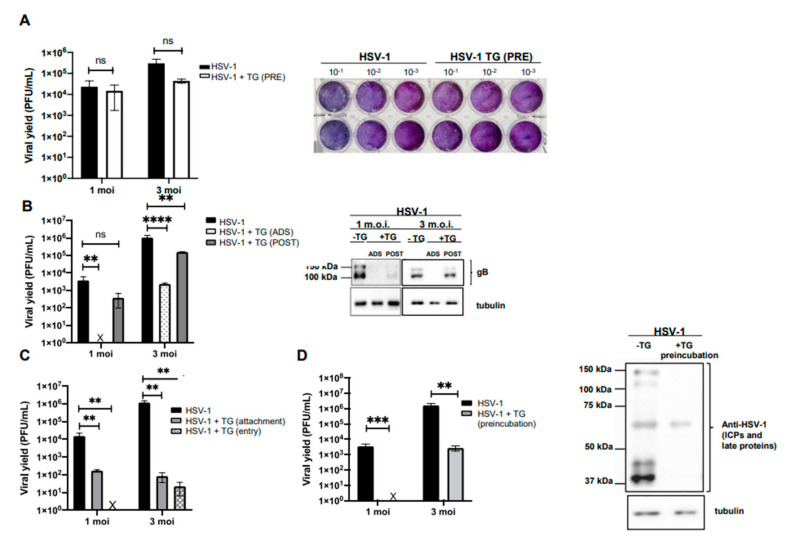
TG reduces HSV-1 replication and exerts a virucidal effect. (**A**) Vero cells were pretreated with TG (50 µg/mL; PRE) for 3 h at 37 °C before HSV-1 infection. Conditioned medium samples were collected 20 h after infection and subjected to SPA (left). Data are mean values ± the SD from four independent experiments, each performed in duplicate (n.s.: not significant; unpaired *t*-test.) For plaques stained with crystal violet, the results of one representative experiment are shown (right). (**B**) A time-of-addition assay was performed by adding TG (50 µg/mL) to Vero cells during viral adsorption to host cells (ADS) or after adsorption for the next 20 h (POST). Supernatants collected following infection and treatment were subjected to SPA to evaluate viral production, expressed in the graph as PFU/mL (left). Data are mean values ± the SD from three independent experiments, each performed in duplicate (**, *p* < 0.01; ****, *p* < 0.0001 versus the untreated sample). Cell lysates were analyzed by western blotting (right) with anti-HSV-1 gB antibody; tubulin was used as a loading control. (**C**) Virus titers of conditioned media from attachment and entry assays calculated as PFU/mL after plaque reduction assays. Data are mean values ± the SD from three independent experiments, each performed in duplicate (**, *p* < 0.01 versus the untreated samples; one-way ANOVA + Dunnett’s multiple comparisons test). (**D**) HSV-1 (1 and 3 MOI) was preincubated with TG at 37 °C for 1 h, and the mixture was used to infect Vero cells for 20 h. Supernatants were collected, and the viral titer was quantified as PFU/mL by SPA (left). Data are mean values ± the SD from three independent experiments, each performed in duplicate (**, *p* < 0.01; ***, *p* < 0.001 versus the untreated sample; unpaired *t*-test). Cell lysates were analyzed by western blotting (right) with an anti-HSV-1 antibody that recognizes viral ICPs and late proteins; tubulin was used as a loading control. A representative experiment is shown.

Firstly, we evaluated whether the antiviral activity of the peptide observed during the time-of-addition assays was due to its interaction with the cytoplasmic membrane of the host cell and, more specifically, with the membrane receptors used by the virus for its attachment. To this aim, Vero cells were pre-incubated with 50 μg/mL TG for 3 h at 37 °C. Cells were then infected with different amounts of HSV-1 (1 and 3 MOI) and the efficacy of the infection was checked by the SPA of cell supernatants harvested 20 h PI. The results demonstrated that the TG pre-treatment of cells does not affect HSV-1 infection (Figure 2A), suggesting that the peptide may act on the virus particles and/or on the first step of its life cycle, rather than on the host cell surface.

Then, to evaluate whether the anti-herpetic property of TG was dependent on the number of viral particles, the peptide (50 μg/mL) was administered under ADS or POST conditions to cell monolayers infected with different amounts (1 and 3 MOI) of HSV-1 (Figure 2B). Under both experimental conditions, the viral titer reduction was about 4 log and 1 log in ADS and POST treatments, respectively (Figure 2B, left panel). These results were confirmed by the decreased expression level of the viral glycoprotein B (gB) in cells treated with TG, as indicated by western blot analysis (Figure 2B, right panel).

To evaluate whether TG specifically inhibits HSV-1 binding to the host cell or its entry, virus attachment and entry assays were performed as described in the Materials and Methods section. The efficacy of the infection was estimated by a plaque reduction assay and quantified as PFU/mL. As shown in Figure 2C, plaque formation appeared reduced under both experimental conditions, but the major reduction was observed during the entry assay, i.e., 3-log viral titer inhibition at both 1 and 3 MOI, although a 1 log reduction was detected during the attachment test. Overall, these results indicate that TG affects HSV-1 infection in Vero cells and suggest a possible action of TG on one or more viral glycoproteins involved in both the binding of the virus to the cell membrane and the subsequent penetration into the host cell. 

### 2.2. TG Directly Affects the HSV-1 Virion

Finally, to verify whether TG exerted a direct action on the viral particle, similarly to what was already shown for temporin B (TB) [8], HSV-1 (1 and 3 MOI) was pre-incubated with 50 μg/mL TG for 1 h at 37 °C. Confluent monolayers of Vero cells were then infected with the HSV-1-TG mixture or with the untreated virus (i.e., not incubated with the peptide) as a control. The potential virucidal effect of TG was evaluated by verifying the efficacy of the infection through SPA on cell supernatants and western blot analysis of total viral protein expression levels in cell lysates harvested 20 h PI. Results demonstrated that virus pre-incubation with TG led to a high and significant inhibition of HSV-1 infection in Vero cells compared to the control (Figure 2D). It was observed that 50 μg/mL TG completely inhibited HSV-1 infection at 1 MOI and partially inhibited infection at 3 MOI, suggesting a direct relation between peptide dose and number of virions.

### 2.3. TG Exerts Slight Antiviral Activity against JCPyV Infection

**Figure 3:**
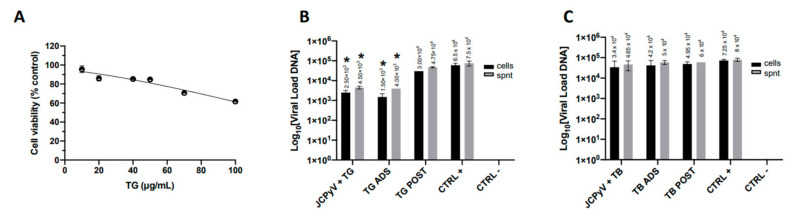
Antiviral properties of TG against JCPyV infection. (**A**) COS7 cells were treated with different TG concentrations to evaluate its cytotoxicity by an MTT assay; (**B**,**C**) time-of-addition assay: 50 µg/mL TG (**B**) or 40 µg/mL TB (**C**) was added to COS7 cells during (ADS) or after (POST) the virus challenge, and viral titers were analyzed in both cell monolayers and conditioned media by Q-PCR 48 h later. Data are mean values ± the SD from two experiments, each performed in triplicate (*, *p* < 0.05; versus the untreated sample (CTRL+); unpaired *t*-test).

Subsequently, we chose to evaluate the effects of the peptide against another neurotropic virus, the naked DNA virus JCPyV, belonging to the Poliomaviridae family. Hence, we firstly evaluated TG cytotoxicity against COS-7 cells, known to be highly permissive to JCPyV [32], by administrating increasing concentrations of TG (from 10 to 100 µg/mL) for 48 h and evaluating cellular proliferation by an MTT assay. Results in Figure 3A show that TG does not affect the viability of COS7 cells at 50 µg/mL, the same concentration used in HSV-1 testing, and its CC_50_ is 129 µg/mL.

Next, COS-7 cells were infected with JCPyV for 48 h, as described in Methods, and treated with TG (50 μg/mL) at different times after infection (a time-of-addition assay): (i) during the adsorption phase of the virus to the host cells (2 h at 37 °C) (ADS); (ii) after 1 h of viral challenge and for the next 48 h (POST). Furthermore, to evaluate the possible effect of the peptide on the virion, the virus was pre-incubated with the peptide for 1 h at 37 °C before using both mixtures to infect cell monolayers. At the endpoints of the infections, cells and supernatants were recovered for the quantification of the JCPyV genome by Q-PCR. The same investigation was performed by using TB (20 μg/mL), another amphibian peptide of the temporin family that is known to exert a virucidal effect on enveloped viruses [8] and is not toxic to COS7, as observed by the trypan blue exclusion assay (data not shown).

The results (Figure 3B) showed that TB does not interfere with JCPyV infection; on the contrary, TG preincubation exerted a slight virucidal effect (1 log reduction) on JCPyV. Similarly, TG administration during virus adsorption to the host cell induced a small but significant reduction of virus infection (1 log). These results suggest that TG may directly alter the JCPyV virion and/or the earliest steps of the virus life cycle.

### 2.4. TG Interacts with the gB Glycoprotein of HSV-1 and with the Capsid Receptor VP1 of JCPyV

To shed light on the molecular binding mechanism of TG to HSV-1 and JCPyV, computational studies were performed. Initially, molecular docking simulations were carried out to investigate the potential interaction of TG with the HSV-1 glycoproteins responsible for the viral adhesion to the cells, i.e., the gB trimer, gD, and the gH-gL complex [33]. Blind docking simulations of TG were thus performed on each of these receptors with the HPEPDOCK [34] program. The best ranked pose obtained for each complex was then optimized by the refinement interface of the HADDOCK suite, which also assigned specific scores and energy terms to the resulting tridimensional models (Table 1) [35]. 

**Figure 4:**
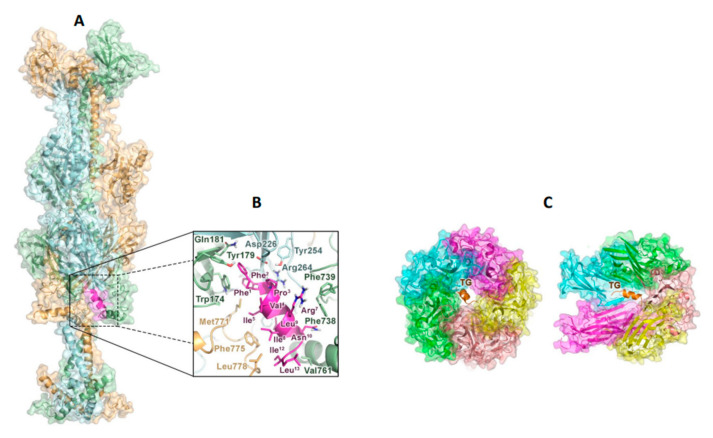
Computational studies indicating putative TG binding with HSV-1 glycoprotein-B and with JCPyV VP1 protein. (**A**) Model complex of the gB trimer and TG: gB is shown as a ribbon and in surface representations (the three monomers are in light orange, light green, and light cyan), while TG is shown as a magenta ribbon. (**B**) TG within the binding site of gB. Side chain of interacting residues are shown as sticks. For the sake of clarity here and throughout the manuscript, the residue numbers of the peptide are reported as an apex, while those of the receptor are not. (**C**) Upper (left panel) and side (right panel) view of the predicted TG/VP1 binding complex. TG is depicted in orange, while the VP1 pentamer subunits are shown in green, cyan, magenta, yellow, and pink and as a transparent surface. To gain insights into the mechanism of action of TG in the JCPyV fusion process, the same docking protocol was applied to investigate the possible interaction of the peptide with the main viral capsid protein, VP1. Docking results indicate that TG can recognize VP1, albeit with a lower affinity compared to HSV-1 gB (Table 1). According to the obtained model, TG was able to bind VP1 within the upper part of the five-fold symmetry pore (Figure 4C) formed by the pentameric arrangement of VP1 monomers [23,37]. Notably, these predictions are in line with recent research showing that peptides derived from the two minor capsid proteins VP2 and VP3 of polyomaviruses can bind to the same region of VP1 [38].

The TG/gB complex turned out to be significantly more stable than the other complexes and was thus considered for further analysis. The docking-predicted TG/gB model shows that TG can bind a membrane-proximal hydrophobic groove at the trimer interface, mainly defined by the fusion loops (residues 173–179 and 258–265) and the helix forming the membrane proximal region (MPR, residues 754–770) (Figure 4A). Here, the peptide can establish numerous nonpolar contacts through its hydrophobic residues (Figure 4B), in line with the high van der Waals contribution to the stabilization of the complex (Table 1). Phe^1^ and Phe^2^ of TG can be hosted in an aromatic cage lined by residues such as Trp^174^ and Tyr^179^ of a first gB monomer (hereafter referred as A). Interestingly, these residues have been previously reported to play a key role during the HSV-1 fusion process [36]. Additional lipophilic interactions can be formed bythe Ile^5^, Ile^8^, and Leu^9^ of TG with residues such as Met^771^, Leu^775^, and Leu^778^ on a second gB unit and by Ile^12^ and Leu^13^ with Val^761^ on the third gB monomer. Finally, cation–π interactions formed by Arg7 with Phe^738^ and Phe^739^ on the A subunit can also contribute to stabilizing the peptide’s binding mode.

## 3. Discussion

In the present paper, we demonstrated the antiviral properties of TG against two neurotropic and widely distributed DNA viruses such as HSV-1 and JCPyV. We focus our attention on these two viruses, because, apart from their different structural peculiarities such as the presence (HSV-1) or absence (JCPyV) of the envelope, they can establish life-long latent infections in humans with periodic reactivations that cause asymptomatic virus shedding or mild to very severe diseases, especially when the viruses reach the CNS, as for HSE and PML. Neither a cure to eradicate the viruses from the host nor a vaccine to prevent their infection is available, but many antiviral drugs have been developed to inhibit virus replication in host cells. However, there is still an urgent need for effective and clinically relevant antiviral compounds that, on one hand, can inhibit specific viral processes without having effects on cells and organs and, on the other hand, can reach the CNS in concentrations sufficient to reduce or prevent diseases related to both HSV-1 and JCPyV. In line with this context, our data provide interesting information for the development of new antiviral strategies.

Herein, we report that TG was effective against HSV-1 infection in a dose-dependent manner, especially when added during the viral challenge. TG efficacy was maintained even when the virus was added at high MOI, indicating a strong impairment of specific steps of viral infection. The attachment and entry assays further confirmed that TG was able to interfere with both steps, with the entry process being affected to a larger extent (4 log inhibition at 1 MOI). These data are in line with what we previously found for respiratory virus infection. We indeed reported that TG impairs influenza virus infection by inhibiting its early life-cycle phases inside host cells, such as those mediated by the viral hemagglutinin (HA) protein [9]. Specifically, our in vitro and molecular docking studies evidenced the formation of a TG/HA complex that prevents the conformational rearrangements of the HA2 subunit, an event essential for the viral envelope fusion with intracellular endocytic vesicles [39]. 

Here, our computational studies support the hypothesis that TG can inhibit the HSV-1 fusion process by interacting with the gB glycoprotein on the viral surface. In particular, molecular docking simulations predicted that TG can recognize a hydrophobic groove formed by the fusion loops, where the peptide can establish key contacts with Trp174 and Tyr179, and the membrane proximal region (MPR) of the gB trimer. Notably, these subdomains are supposed to play a key role in HSV-1 infection, either by triggering the activation of gB itself or by facilitating lipid mixing [36,40]. Altogether, these outcomes indicate that, by interacting with specific gB regions, TG can impair the viral fusion machinery. Nonetheless, since the peptide has demonstrated a significant virucidal activity, we cannot exclude that it might also affect the function of other glycoproteins such as gH-gL or gD, which would further inhibit the cell penetration of the virus.

Furthermore, we observed a virucidal effect of TG against HSV-1, comparable to that of TB [8]. As indicated by circular dichroism spectroscopy [9], both peptides adopt an α-helix structure in environments that mimic cell membranes or the viral envelope, suggesting that TG, similarly to TB, can alter the permeability of the viral envelope causing its degradation. 

We also found that TG impairs the early steps of the JCPyV life cycle, possibly by interacting with the viral protein VP1, as supported by both in vitro assays and docking simulations. These data suggest that the peptide can bind to the upper region of the 5-fold pore formed by the major capsid component VP1 [23]. Interestingly, recently developed peptides, endowed with effective anti-polyomaviruses properties, were found to bind to the same site on VP1 [38]. In line with this evidence, it has been reported that ligand-induced structural perturbations occurring at the VP1 pore can significantly impair the JCPyV entry process [37], thus explaining the efficacy of TG against this virus.

Remarkably, previous papers reported that AMPs belonging to the human Defensin family can counteract JCPyV infection by affecting its earliest steps in the virus life cycle. This indicates that other AMPs of human or animal origin can alter JCPyV infection [41,42]. 

According to our data, further studies to verify TG efficacy in in vivo models of infection are urgently required. In particular, testing the peptide’s effectiveness against virus-related brain diseases should be a top priority if TG brain distribution is to be confirmed. Several in vivo models of HSV-1 infection have been developed, including those suitable for studying the long-term effects of recurrent infection in the brain [43,44]. However, in vivo models for cerebral JCPyV-related diseases are currently not available. Nevertheless, a human-derived brain organotypic system has been recently developed to model virus infection and virus-induced brain pathologies [45,46]. 

In conclusion, the present study paves the way for the potential development of short naturally occurring peptides as novel agents aimed at reducing virus reactivations and the emergence of drug resistance in immunocompromised patients, who require longer-term therapy. 

## 4. Materials and Methods

### 4.1. Peptides

Synthetic TG (F1F2P3V4I5G6R7I8L9N10G11I12L13-NH2) and TB (L1L2P3I4V5G6N7L8L9K10S11L12L13-NH2) peptides were purchased from Biomatik (Wilmington, DE, USA). Each peptide was synthesized by solid-phase Fmoc chemistry methodology. A purity of 95% was obtained via reverse-phase high-performance liquid chromatography (RP-HPLC) using a gradient of 0.1% trifluoroacetic acid in acetonitrile from 20% to 100% in 30 min at a flow rate of 1 mL/min (Gemini 5 µm C18 110A HPLC Column 250 × 4.6 mm). The molecular mass of the peptides was verified by mass spectrometry. All peptides were dissolved in water; stock solutions of 2 mM were prepared.

### 4.2. Cell Cultures

African green monkey (Vero) cells (ATCC CCL-81™) were grown at 37 °C in an atmosphere of 5% of CO2 in an RPMI 1640 medium supplemented with 10% heat-inactivated fetal bovine serum (FBS), 0.3 mg/mL L-glutamine, 100 U/mL penicillin, and 100 µg/mL streptomycin (Sigma-Aldrich S.r.l., Milan, Italy). COS-7 cells were obtained from the ATTC (ATCC^®^ CRL-1651TM). COS-7 is a derivative of CV-1 (ATCC^®^ CCL-70TM), a cell line established from the kidney of an African green monkey that was transformed with an origin-defective mutant of SV40 [47]. Dulbecco’s modified Eagle medium (DMEM) supplemented with 100 U of penicillin, 100 μL of streptomycin per mL (Sigma-Aldrich S.r.l., Milano, Italia), and heat-inactivated FBS (10%) was used as a maintenance medium for the cell line. The cells were incubated at 37 °C in the presence of 5% CO_2_.

### 4.3. HSV-1 and JCPyV Virion Production

HSV-1 production was performed as previously reported [48]. Briefly, monolayers of Vero cells in 75 cm^2^ tissue culture flasks were infected with HSV-1 (strain F) at 0.01 MOI. After 48 h at 37 °C, the time required to observe the virus-induced cytopathic effect, infected cells were harvested with 3 freeze-and-thaw cycles, cellular debris was removed with low-speed centrifugation for 10 min, and the virus titer was measured on the supernatants by SPA, as described below. The titer of the virus preparation was 5 × 10^8^ PFU/mL. The virus was stored at −80° C until used. 

JC virion production was obtained as previously reported [47]. Briefly, COS-7 cells (7 × 10^4^) were grown for 24 h in a complete medium to reach 50–70% confluence on the day of transfection. The cells were then transfected with 1 μg of JCPyV CY strain DNA following the specifications of the Xfect TM Transfection Reagent kit (Clontech Laboratories, Inc., Mountain View, CA, USA), incubated at 37 °C for 4 h with the transfection mixture. After two washes with phosphate-buffered saline (PBS), cells were incubated with a complete culture medium for the time-course experiment. After two days of incubation, COS-7 cells were transferred to a 34 mL flask and continuously cultured in the maintenance medium. Once a week for 35 days, supernatants were harvested, subjected to 6 cycles of freezing and thawing and centrifuged at 2000 rpm for 5 min, and the resulting clarified supernatants were quantified by Quantitative Real-Time PCR (Q-PCR) assay, as described below. Stocks of supernatants containing virions corresponding to 1 × 10^5^ equivalent genomes per milliliter (gEq/mL) were stored at −80° C until used.

### 4.4. Cellular Toxicity Assays

The cellular toxicity of TG was evaluated by a Trypan blue (0.02% final concentration) exclusion assay [49]. The viability and growth of Vero cells were tested in vitro by an MTT [3-(4, 5-dimethylthiazol-2-yl)-2, 5-diphenyl tetrazolium bromide] assay, as previously reported [50]. Briefly, cellular monolayers were incubated with the peptide at concentrations of 10, 20, 40, 50, 70, and 100 µg/mL (corresponding to 6.9, 13.7, 27.4, 34, 48, and 68 µM, respectively) in a culture medium for 24 h; the medium was replaced with 10 µL of a 1 mg/mL solution of an MTT medium without phenol red (Sigma-Aldrich). Cells were incubated at 37 °C for 2 h, and 100 µL of acidified isopropanol (HCl 0.1N in isopropanol) was then added to each well. After a few minutes at room temperature to ensure that all crystals were dissolved, the plates were read using an automatic plate reader with a 570 nm test wavelength and a 690 nm wavelength. The CC_50_, defined as the drug concentration required to reduce the cell viability by 50%, was calculated by regression analysis of the dose–response curve generated from the data.

### 4.5. HSV-1 Infection and Determination of Viral Yields

Vero cells were seeded in 24-well plates at a density of 1.3 × 10^5^ cells/mL and infected with HSV-1 at 1 or 3 MOI. After incubation for 1 h at 37 °C to allow virus adsorption to the host cells (viral challenge), the medium was removed and replaced with a fresh medium supplemented with 2% FBS. The plates were then maintained for 20 h at 37 °C in an atmosphere of 5% CO_2_. The viral titer was measured through SPA, as previously described [51]. Briefly, supernatants of infected samples were used at different dilutions to infect the monolayers of Vero cells. After 1 h at 37 °C, the monolayers were washed, the medium was replaced with RPMI containing 2% carboxymethylcellulose (CMC) and 2% FBS, and the plates were maintained at 37 °C for 48–72 h until plaque formation. Thus, the CMC solution was removed, and the monolayers were fixed with cold methanol for 20 min at −20 °C, washed with PBS, and stained with a 0.5% crystal violet solution. The plaques were counted, and the viral titer was calculated as PFU/mL.

### 4.6. JCPyV Infection and Quantification

COS-7 cells, (5 × 10^5^) were plated at a density of 5 × 10^5^ and grown in a complete medium to reach 50–70% confluence on the day of infection. After 24 h, cells were infected with supernatant harvested from a previous transfection experiment and containing virions corresponding to 1 × 10^5^ gEq/mL. After a 2 h adsorption phase, the cells were washed twice with PBS and incubated with a fresh medium for 24 h. At the end of the infection, total DNA was extracted from 1 × 10^6^ cells using the QIAmp^®^ DNA Mini Kit (QIAGEN S.p.A, Milan, Italy), following the instructions provided by the manufacturer. Once extracted, the DNA was stored at −20 °C until the use. The supernatant (1 mL), recovered from infected cells, was boiled for 10 min and centrifuged at 2000 rpm for 10 min [52]. The resulting clarified supernatants were used directly for DNA quantification.

The Q-PCR assay was performed on all the extracted DNA samples and on the supernatants using a 7300 Real-Time PCR System (Applied Biosystems, Waltham, MA, USA), following a published protocol [47,53]. The amplification data were analyzed using SDS 1.4 software (AB Applied Biosystems, Foster City, CA, USA).

Each sample was analyzed in triplicate, and the viral loads were given as the mean of at least three positive reactions. Standard precautions to prevent contamination were followed, and a negative control (NTC) was included in each run. Viral DNA was quantified using a standard curve consisting of serial dilutions of a plasmid containing the entire JCPyV genome with a known titer (range, 10^5^ gEq/mL–10^2^ gEq/mL). The amount of cellular DNA was quantified simultaneously using a SYBR GREEN PCR for the house- keeping β-globin gene [54] and used to normalize the JCPyV DNA.

### 4.7. Plaque Reduction Assay

To assay the anti-HSV-1 activity of TG, a plaque reduction assay was performed as previously described, with some modifications [8]. Vero cells were seeded in 24-well plates at a density of 1.3 × 10^5^ cells/well. The next day, confluent cell monolayers were infected with HSV-1 at 1 or 3 MOI for 1 h at 37 °C. The unabsorbed virus was subsequently removed by washing the cells three times with PBS, and the medium was replaced with RPMI 1640 containing 1% CMC, 2% FBS, and different concentrations of the peptide. After further incubation at 37 °C for 36/48 h, cells were fixed with cold methanol and stained with a 0.5% crystal violet solution, and viral plaques were counted. The concentration causing a 50% reduction in plaque formation (IC_50_) was determined by regression analysis using Graph Pad Prism version 6.0 software by fitting a variable slope-sigmoidal dose–response curve.

### 4.8. Pre-Treatment Assay

Vero cells were incubated with 50 µg/mL TG for 3 h at 37 °C, washed with PBS, and infected with HSV-1 at 1 or 3 MOI for 1 h. Cells were maintained in a fresh medium supplemented with 2% FBS for the following 24 h. Subsequently, supernatants of infected cells were used to determine the HSV-1 titer by a standard plaque assay. 

### 4.9. Time-of-Addition Assay 

TG (50 μg/mL) was added to a confluent monolayer of Vero cells infected with HSV-1 (1 or 3 MOI) at different times of infection, as follows: during viral challenge (1 h at 37 °C), immediately after the adsorption period, and continuously for the following 20 h. The supernatants from the infected cells were used to determine the virus titer by SPA, as described above. TB (20 μg/mL) or TG (50 μg/mL) was added to a confluent monolayer of COS-7 cells infected with JCPyV (1 × 10^5^ gEq/mL) at different times after infection: during viral challenge (2 h at 37 °C), immediately after the adsorption phase, and continuously for the following 20 h. Cells and supernatants were recovered for the quantification of the JCPyV genome by Q-PCR, as described above.

### 4.10. Attachment Assay

The attachment assay was performed as previously described [55] with some modifications. Briefly, Vero cells were incubated with a combination of TG (50 μg/mL) and HSV-1 (1 or 3 MOI) at 4 °C for 1 h. Cells were then washed twice with PBS to remove both the unattached virus and peptide and maintained for 1 h at 37 °C in RPMI (adsorption phase). Following three more washes with PBS, cells were incubated at 37 °C with RPMI 1640 containing 1% CMC and 2% FBS to allow the formation of plaques. Monolayers were then fixed with cold methanol and stained with a 0.5% crystal violet solution, and plaques were counted to determine the viral titer.

### 4.11. Entry Assay

The entry assay was performed as previously reported [56] with some modifications. Briefly, confluent monolayers of Vero cells were infected with HSV-1 (1 or 3 MOI) at 4 °C for 1 h. Cells were then incubated at 37 °C to maximize the penetration of virus in the presence or absence of TG (50 μg/mL). After 1 h, the supernatants were removed, and the cells were treated with acidified PBS (pH 3) for 1 min to neutralize any remaining attached virus. After four washes with serum-free RPMI 1640, cells were overlaid with a medium containing 1% CMC and 2% FBS and incubated at 37 °C to allow plaque formation. Monolayers were then fixed with cold methanol and stained with a crystal violet solution, and plaques were counted to determine the viral titer.

### 4.12. Virucidal Activity 

TG (50 μg/mL) was pre-incubated with HSV-1 (1 or 3 MOI) at 37 °C for 1 h, and the mixture was used to infect cellular monolayers. Following viral challenge, the plates were washed, and the medium was replaced with a fresh medium containing 1% CMC and 2% FBS for 20 h at 37 °C. Supernatants were then recovered, and a viral titer was evaluated as already described. 

TB and TG were pre-incubated with JCPyV (1 × 10^5^ gEq/mL) for 1 h at 37 °C, and both mixtures were used to infect COS-7 cell monolayers. After 2 h of viral adsorption, cell monolayers were washed with PBS and incubated in a complete medium supplemented with 2% FBS for 48 h at 37 °C. Cells and supernatants were then recovered for the quantification of the JCPyV genome by Q-PCR, as described above.

### 4.13. Western Blot Analysis

HSV-1-infected Vero cells, treated or untreated with TG, were washed with PBS, recovered, and centrifuged at 770× *g* for 10 min. The pellet was suspended in a cold lysis buffer (10 mM Tris-HCl, 150 mM NaCl, and 1 mM phenylmethylsulfonyl fluoride (PMSF), supplemented with phosphatase and protease inhibitor mixtures [Sigma] as well as 1% Triton X-100, pH 7.4) and incubated for 30 min on ice. After centrifugation (13,000× *g* for 30 min at 4 °C), the supernatants were collected and assayed to determine their protein concentration (Bradford method, Bio-Rad). Equivalent amounts of proteins were separated by SDS-PAGE and blotted onto nitrocellulose membranes for the western blot assay. The membranes were blocked with 10% nonfat dry milk (Biorad) in TBS-Tween 0.1% for 1 h at room temperature. Primary antibodies (anti-glycoprotein B, Santa Cruz Biotecnology, Dallas, TX, USA; anti-HSV-1, Biorad, Herakles, GA, USA; anti-tubulin, Sigma, St. Louis, MI, USA) were added at a final concentration of 1 μg/mL and maintained overnight at 4 °C. Horseradish peroxidase-conjugated antibodies were used as secondary antibodies (Jackson ImmunoResearch, West Grove, PA, USA). Blots were developed with an ECL-Plus Detection System (Thermo Scientific, Waltham, MA, USA). 

### 4.14. Modeling of the Complexes of HSV1 and JCPyV Selected (Glyco)Proteins with TG

The models of the complex between the TG peptide and the different (glyco)proteins of HSV-1 or JCV involved in the fusion (gD, gH-gL complex, and gB for HSV1; VP1 for JCPyV) were obtained by performing docking calculations. Information about the available structural data for gD (pdb code 3U82) [57], gB (pdb code 5V2S) [40], and VP1 (pdb code 3NXD) [23] was retrieved and downloaded from the Protein Data Bank (www.pdb.org; accessed on 23 March 2021). Missing loops in the gB crystal structure were modeled using Maestro (Schrödinger, LLC, New York, NY, USA, 2021). Models of the gH and gL proteins were generated starting from the crystal structures of the corresponding proteins in HSV-2 (pdb code 3M1C) [58] using the I-TASSER server for the protein structure and function prediction, which is based on a threading alignment algorithm [59]. The gH-gL complex was generated by manual superposition with the same complex of HSV-2 and refined using the Refinement Interface of the web server HADDOCK 2.2 [35]. Input coordinates for the TG peptide were generated as recently reported [9]. 

Docking calculations were performed with HPEPDOCK software (version 2021-11-13) [34]. HPEPDOCK is an innovative online server for investigating protein-peptide docking based on the hierarchical algorithm. It performs a blind and flexible peptide docking by the fast modeling of peptide conformations and the sequent global sampling of binding orientations. The best model, obtained by HPEPDOCK for each complex (TG/gD, TG/gH-gL, TG/gB, and TG/VP1) was subjected to refinement using the Refinement Interface of the web server HADDOCK 2.2 and scored according to the HADDOCK-scores [35]. The Refinement Interface protocol consists of three stages: (i) a rigid body energy minimization that generates 20 structures, (ii) a semi-flexible refinement in torsion angle space, and (iii) a refinement in Cartesian space with an explicit solvent. The Refinement Interface of HADDOCK 2.2 generates a result (HADDOCK score) based on a weighted sum of Van der Waals, electrostatic, and desolvation energies as well as buried surface area values in combination with AIR energy, which has been used as criteria in the selection of the best complex. Energy terms of the water-refined HADDOCK models are reported in Table 1.

### 4.15. Statistical Analysis

Normally distributed data comprised of two groups was assessed using the Student’s *t*-test. Where three or more groups were present, one-way ANOVA and Dunnett’s multiple comparisons test were utilized, as detailed in the figure legends. *p* values < 0.05 were considered significant. All data are presented as mean ± standard deviation (SD) of *n* = 2 or *n* = 3 of independent experiments (SD). 

## Figures and Tables

**Table 1:** Lowest energy pose HADDOCK refinement outputs.

Complex	H Score ^a^	RMSD ^b^	VdW ^c^	Eletr ^d^	Desolv ^e^	Restr ^f^	BSA ^g^
gB/TG	−178.0 ± 9.7	0.3 ± 0.2	−66.7 ± 2.1	−27.9 ± 13.0	−105.8 ± 10.4	0.9 ± 0.2	1795 ± 14
gD/TG	−93.2 ± 1.6	0.3 ± 0.2	−51.0 ± 2.3	−48.9 ± 6.3	−32.5 ± 1.0	0.0 ± 0.0	1200 ± 21
gH-gL/TG	−93.9 ± 1.6	0.3 ± 0.2	−72.6 ± 2.8	−21.6 ± 5.4	−17.0 ± 2.1	0.2 ± 0.2	1714 ± 48
VP1/TG	−48.7 ± 6.6	0.2 ± 0.1	−70.7 ± 1.3	−62.0 ± 10.8	33.8 ± 5.3	5.4 ± 0.9	2134 ± 13

^a^ HADDOCK score. ^b^ RMSD from the overall lowest-energy structure. ^c^ Van der Waals energy. ^d^ Electrostatic energy. ^e^ Desolvation energy. ^f^ Ambiguous Interaction Restraint (AIR) energy. All terms are given in kcal/mol. ^g^ Buried surface area (Å2).

## Data Availability

All relevant data are within the manuscript.
